# New Insights into the Stereochemical Requirements of the Bombesin BB1 Receptor Antagonists Binding

**DOI:** 10.3390/ph13080197

**Published:** 2020-08-17

**Authors:** Bahareh Rasaeifar, Patricia Gomez-Gutierrez, Juan J. Perez

**Affiliations:** Department of Chemical Engineering, Universitat Politecnica de Catalunya, ETSEIB, Av. Diagonal, 647, 08028 Barcelona, Spain; bahareh.rasaeifar@upc.edu (B.R.); ondopasa@gmail.com (P.G.-G.)

**Keywords:** bombesin receptors, neuromedin B antagonism, G-protein coupled receptors homology modeling, non-peptide neuromedin B antagonists

## Abstract

Members of the family of bombesinlike peptides exert a wide range of biological activities both at the central nervous system and in peripheral tissues through at least three G-Protein Coupled Receptors: BB1, BB2 and BB3. Despite the number of peptide ligands already described, only a few small molecule binders have been disclosed so far, hampering a deeper understanding of their pharmacology. In order to have a deeper understanding of the stereochemical features characterizing binding to the BB1 receptor, we performed the molecular modeling study consisting of the construction of a 3D model of the receptor by homology modeling followed by a docking study of the peptoids PD168368 and PD176252 onto it. Analysis of the complexes permitted us to propose prospective bound conformations of the compounds, consistent with the experimental information available. Subsequently, we defined a pharmacophore describing minimal stereochemical requirements for binding to the BB1 receptor that was used in silico screening. This exercise yielded a set of small molecules that were purchased and tested, showing affinity to the BB1 but not to the BB2 receptor. These molecules exhibit scaffolds of diverse chemical families that can be used as a starting point for the development of novel BB1 antagonists.

## 1. Introduction

Members of the bombesinlike family of peptides, originally isolated from the skin of diverse amphibians and later found to be widely distributed in mammals [1,2], are compounds with a wide spectrum of biological activity. Thus, in the central nervous system they are involved in satiety, control of circadian rhythm and thermoregulation, whereas in peripheral tissues, stimulation of gastrointestinal hormone release, macrophages activation and effects on development [3]. In addition, they are known to play a role in the control of cellular proliferation [4,5]. Actions of this family of peptides are mediated through at least three G-Protein Coupled Receptors: the neuromendin B receptor (known as BB1R), the gastrin-releasing peptide receptor (known as BB2R), and the orphan, since its endogenous ligand has not been disclosed yet, bombesin receptor subtype 3 (known as BB3R) [6,7,8]. Due to the wide spectrum of biological activities mediated by these receptors, there is considerable interest in understanding their potential use as therapeutic agents. A literature review reveals potential therapeutic use for both agonists and antagonists targeting any of the three receptors for cancer therapy [9,10]: BB3R agonists for the treatment of obesity/diabetes mellitus [11]; BB2R antagonists for the treatment of radiation-induced lung injury [12] and BB1R or BB2R antagonists for the treatment of itching in atopic dermatitis [13].

**Figure 1:**
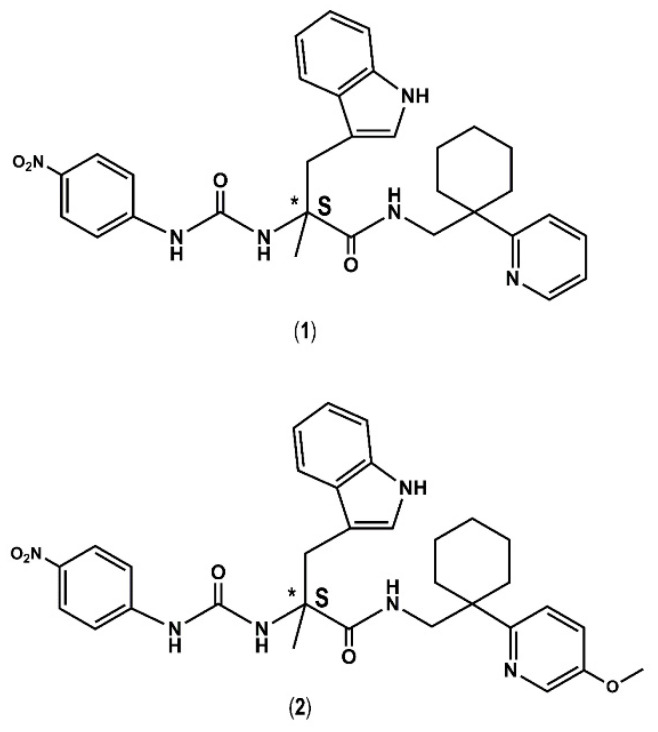
Chemical structures including the chirality of the asymmetric carbon (*) of the bombesin antagonists studied in the present work. PD168368 (**1**) and PD176252 (**2**).

Neuromedin B (NMB) and the gastrin-releasing peptide (GRP), together with its shorter version GRP(18–27)—known as neuromedin C (NMC)—are the mammal endogenous ligands of the BB1R and BB2R, respectively [1,2]. They are selective agonists for the respective receptors, binding with high affinity: NMB exhibits a Ki = 0.052 nM for the BB1R and about 1000 times higher for the BB2R, whereas GRP exhibits a Ki = 0.19 nM for the BB2R and about 1000 times higher for the BB1R [2]. Neither NMB nor GRP bind to the BB3R, and although its endogenous ligand has not been disclosed, several selective peptide and nonpeptide agonists have been disclosed [2]. On the other hand, a few peptide antagonists with a diverse degree of selectivity have also been disclosed for the three receptors [2,14,15]. However, the poor oral bioavailability, low absorption, rapid degradation by proteolytic enzymes and immunogenic profile of peptides make nonpeptide molecules more desirable [16,17]. Efforts in this direction resulted in the discovery of second generation peptoids PD168368 and PD176252 (Figure 1) [18], along with a set of analogs with diverse substitutions [19] that exhibit an antagonist profile for the BB1R and BB2R, with diverse degrees of selectivity. Specifically, PD168368 exhibits a Ki = 0.5 nM for the BB1R and 1700 nM for the BB2R, whereas PD176252 exhibits a Ki = 0.5 nM for the BB1R and 170 nM for the BB2R [2]. Furthermore, the same scaffold was also later used to design ML-18, a BB3R selective antagonist [20], and more recently, compounds AM-37 and ST-36 with diverse pharmacological profile to the diverse bombesin receptors [21]. However, the similarity between the chemical structures of these compounds is so big that it makes it intriguing to understand the subtle differences that provide selectivity for the different receptors. In the absence of ligand–receptor 3D complex structures, rationalization of the structure–activity needs to be carried out indirectly, although the lack of structural diversity of the known binders makes it a difficult job. Complementary to the structure–activity studies, it was pointed a few years ago in an interesting report [22] that Tyr220 in BB1R (corresponding to Phe218 in BB2R) can explain the differential behavior exhibited by PD168368 for the two receptors.

Despite knowledge accumulated in the past to ascertain the potential use of bombesin antagonists as therapeutic agents, advancement has been hindered because of the low diversity within the ligands available. Moreover, PD168368 and PD176252 have been reported to be potent agonists of the human formyl-peptide receptors, questioning the interpretation that their reported effects can be solely attributed to their activity as BB1/BB2 antagonists [23]. Only a few compounds devoid of a peptoid scaffold have been disclosed in the literature to date: a BB1R antagonist with a dibenzodiazepine scaffold [24], the reported antagonist of the BB2 receptor NSC-77427 [25] and Bantag-1, a peptidomimetic designed by an isostere replacement that exhibits a selective antagonist profile for the BB3R [26]. In order to have a better understanding of the therapeutic potential of bombesin antagonists, there is a need to discover novel compounds based on different chemical structures. In this direction, we report, in the present work, the results of a molecular modeling study aimed at discovering novel chemical scaffolds suitable to develop novel bombesin antagonists. For this purpose, we constructed a 3D model of the BB1 receptor by homology modeling and docked PD176252 in diverse poses. Analysis of the ligand–receptor complexes together with known structure–activity studies of this family of compounds permitted to define a pharmacophore that was subsequently used for in silico screening. The results of the study permitted the identification of a limited set of small molecules that were purchased and tested at 50 µM for its capacity to antagonize NMB at the BB1R. The results of this study are disclosed in the present report.

## 2. Results and Discussion

**Figure 2:**
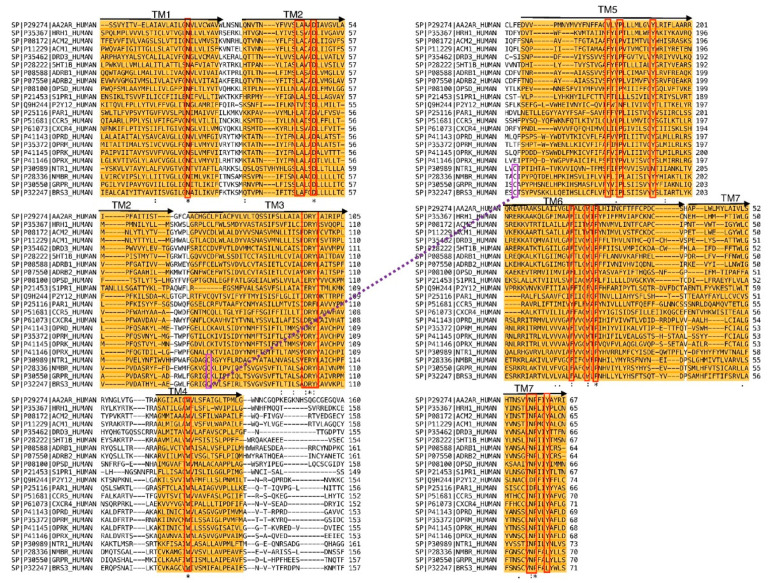
Multiple sequence alignment of diverse GPCRs used in the present work (see text). Transmembrane segments are colored in orange and conserved residues are in red boxes. There is also a purple line indicating a disulfide bridge.

Figure 2 shows the result of the multiple alignment of the diverse sequences of the class-A rhodopsin family used in the present work. As can be seen, the alignment was robust enough to show properly aligned diverse conserved motifs, specifically, a set of conserved residues including (in the Ballesteros-Weinstein numbering scheme [27]) N1.50 in TM1; L2.46, A2.47, D2.50 in TM2; D/E3.49, R3.50, Y3.51 in TM3; W4.50 in TM4; F5.47, P5.50, Y5.58 in TM5; F6.44, W6.48, P6.50 in TM6; N7.49, P7.50, Y7.53 in TM7. Focusing on the sequences of the NTS1 and BB1 receptors, the conserved residue Y5.58 in the former was an N5.58, but this difference did not induce any structural consequence. In contrast, Y7.53 in the former corresponded to Y7.54 in the latter. A closer inspection of the alignment suggests that this corresponded to a displacement and not an insertion that could induce a buldge in TM7. Other motifs such as D(E)RY in TM3, CWxP(Y/F/L) in TM6 or the NPxxY in TM7 were well aligned.

**Figure 3:**
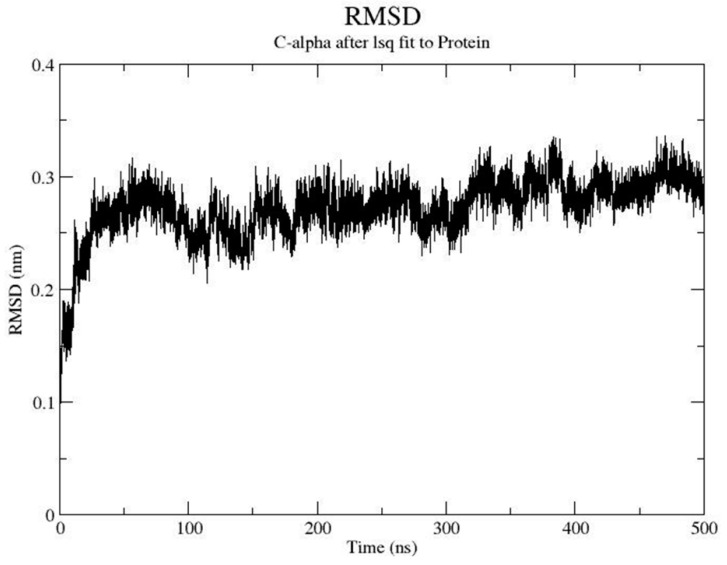
Time evolution of the root mean square deviation (rmsd) of the bombesin BB1 receptor during the refinement process. The rmsd was computed using the alpha carbons of the protein.

Figure 3 shows the evolution of the root-mean-square-deviation (rmsd) computed using the Cα of the BB1 receptor structure along the MD trajectory. A can be seen, it required more than 100 ns for the structure to have the structure equilibrated as had been observed previously with other MD simulations of GPCRs [28]. The procedure permitted the construction of a final model of the BB1 receptor that was generated as the average structure obtained using the last 100 ns of the molecular dynamics trajectory. This structure was subsequently minimized in a two-step procedure, using the steepest descent method with a distance dependent dielectric constant of **2**. First, side chains were optimized with the backbone atoms constrained to be subsequently released in a second minimization step.

In order to understand the molecular features that ligands need to bind onto the BB1 receptor, we analyzed the complex of PD168368 and PD176252, respectively, in their prospective bound conformations. For this purpose, we exploited the available structure–activity studies [18,19], the available results from mutagenesis studies [22] as well as the small residue differences between the BB1R and the BB2R at the orthosteric site. Accordingly, the constructed model of the BB1 receptor was used for docking the antagonists in diverse conformations and orientations. For this purpose, we followed the same protocol as described above. Multiple orientations were obtained that were rank ordered using the XP scoring function of GLIDE [29]. The diverse complexes were subsequently energy minimized in vacuo with a distance dependent dielectric constant of **2**, using the steepest descent method to allow the ligand to adapt to the environment.

Taking the asymmetric carbon of the ligands as reference, the structure of both compounds can be described as composed of three branches including a nitrophenylurea moiety, an indole moiety and a 2-pryridinecyclohexane moiety. The diverse orientations and conformations found permitted the three legs to occupy diverse sites of the orthosteric site of the BB1 receptor and analyze diverse ligand–receptor interactions. As can be expected, the orientations adopted by PD168368 and PD176252 were comparable due to their size and the small structural differences between them. Actually, the difference between the two molecules consisted of an extra methoxyl group at the pryridine moiety in PD176252, as shown in Figure 1. However, this extra moiety provided PD176252 with an order of magnitude improved affinity to the BB2R that can be attributable to an additional favorable interaction with the receptor.

**Figure 4:**
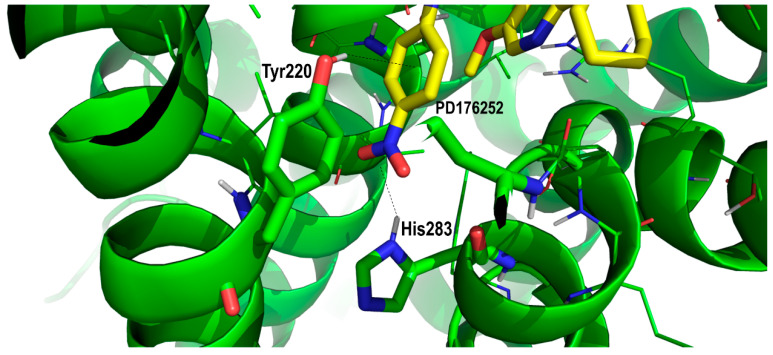
Close-up of PD176252 in its prospective bound conformation to the bombesin BB1 receptor, showing the interaction between its nitrophenyl moiety and diverse residues BB1R including Tyr220 and His286 (see text).

Therefore, in order to assess the most likely binding mode of the ligands to the receptor, we analyzed the diverse ligand–receptor interactions, aimed at the identification of key residues, and checked their conservation in the BB2R to understand if a specific interaction could be responsible for the observed differential binding affinity of the compounds for the two receptors. This assumes that differences in the free energy of binding between PD168368 and PD176252 are attributable only to the enthalpic component. Obviously, differences in the affinity of the two compounds could also be attributed to the ability of the differential chemical group to kick out a bound water molecule from the receptor, improving the affinity of a ligand by an order of magnitude [30]. As mentioned before, early site-directed mutagenesis studies demonstrated the involvement of Tyr220 of BB1 in the binding of PD168368. Actually, when Tyr220 is mutated to a phenylalanine, binding drops to that observed for the BB2R [22]. This result suggests that PD168368, and presumably PD176252, exhibit a critical interaction with Tyr220 in the BB1R bound complex that disappears when the residue is mutated to Phe220. Since the replacement of Tyr for Phe represents a conservative mutation, it can be concluded that the hydroxyl group of the tyrosine is involved in a hydrogen bond with the ligand. This represents an important structural constraint that permits us to discard a few poses from the docking study. Analysis of the diverse poses consistently showed a subset of them with the hydroxyl group of the Tyr220 side chain pointing towards the center of the aromatic ring of the nitrobenzene ring of the ligand, as shown in Figure 4. Interestingly, the interaction between both moieties was reinforced by a quadruple–quadruple interaction between the aromatic ring of the ligand and the aromatic ring of the tyrosine side chain adopting a T-shape relative orientation. This explains in part the lower affinity exhibited by PD168368 and PD176252 for the BB2R. In this case, although the quadrupole–quadrupole interaction between aromatic rings was preserved in the complex with the BB2R, the loss of a hydrogen bond can be associated with an order of magnitude drop of affinity [31]. In addition, the nitro group formed a hydrogen bond with His283 (residue conserved in the BB2R), reinforcing the role played by the nitrophenyl moiety in the affinity of the two ligands, as shown in Figure 4.

**Figure 5:**
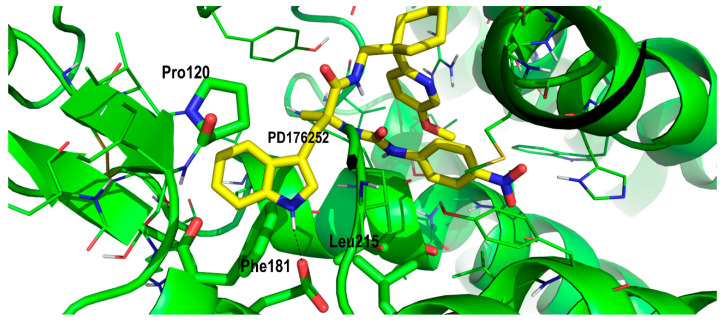
Close-up of PD176252 in its prospective bound conformation to the bombesin BB1 receptor, showing the interaction between its indole moiety and residues Phe181, Pro120 and Leu215 (see text).

The indole moiety in both ligands sat in a hydrophobic pocket defined by residues Phe181, Pro120 and Leu215 (Figure 5). In addition, the indole moiety was oriented in such a way that it formed a hydrogen bond with Glu178. All these residues were conserved in the BB2R, so these interactions help to explain part of the affinity of the ligand, but they do not explain the higher affinity observed by PD168368 and PD176252 towards the BB1R.

**Figure 6:**
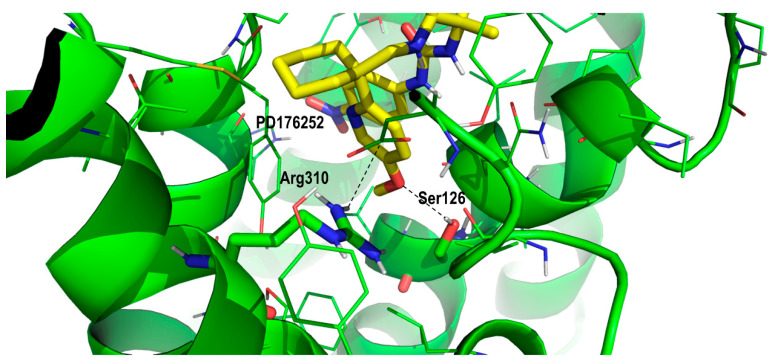
Close-up of PD176252 in its prospective bound conformation to the bombesin BB1 receptor, showing the interaction between its phenoxyl-2-pyridine moiety interacting with diverse residues including Ser126, Gln123 and Arg310 (see text).

**Figure 7:**
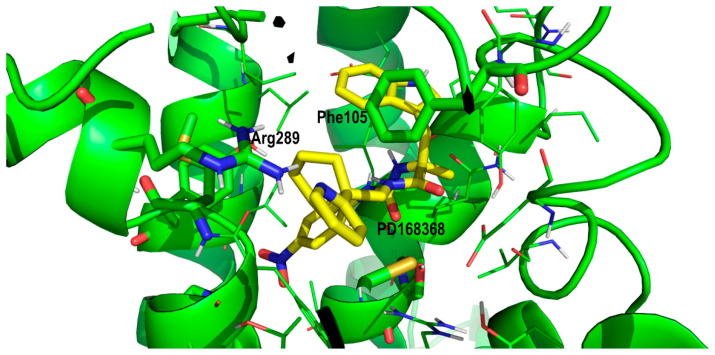
Close-up of PD168368 in its prospective bound conformation to the bombesin BB1 receptor, showing the interaction between its phenoxyl-2-pyridine moiety and diverse residues including Arg289 and Phe105 (see text).

To understand the difference in the binding affinity between PD168368 and PD176252 towards the BB2R we should focus on the role of the methoxyl group attached to the 2-pyridinecyclohexane moiety. As mentioned above, due to the similar size of the ligands, it can be hypothesized that PD168368 and PD176252 adopt a similar bound conformation to the BB1R. Analysis of the diverse poses with the nitrophenyl moiety accommodated in the proximity to Tyr220, revealed a subset of poses, where the methoxypyridinyl group nicely sat in a pocketin which the side chain of Ser126 (residue conserved in the BB2R) is located at a suitable distance to form a hydrogen bond with the oxygen of the methoxyl moiety (Figure 6). In addition, the side chain of Arg310 (also conserved in BB2R) that formed a hydrogen bond with Asp100 in the apo form, was in the position to form a hydrogen bond with the center of the aromatic ring of the PD176252 pyridine ring. The presence of a hydrogen bond between the ligand and Ser126 can explain the affinity difference between PD168368 and PD176252 towards the BB2R. However, it does not explain that the affinity of both compounds to the BB1R remained the same. This can be explained on the basis that the 2-pyridine moiety did not appear to bind in the same conformation in both ligands. In the case of PD168368, the pyridine ring was likely to be accommodated close to Arg289, in such a way that its side chain formed a hydrogen bond with the nitrogen of the heterocycle (Figure 7). Since this residue was conserved in both receptors, this effect was expected to be found in both the BB1R and the BB2R and does not contribute to explaining the affinity difference of the compound for the two receptors. However, the position of the heterocycle permitted an additional interaction with Phe105 via a quadrupole–quadrupole interaction that was not found in the BB2R, since this residue was not conserved (it corresponded to Leu102 in BB2R). In addition, the cyclohexane group accessed a hydrophobic pocket formed by residues Met287 (Leu285 in BB2R), the conserved Ile296 and the aliphatic chain of the conserved Lys210. These differences, together with the differential interaction with Tyr220, can explain the affinity difference of the compounds for the two receptors. Accordingly, the fact that PD176252 exhibited the same affinity for BB1R and BB2R could be explained as a compensation of interactions, in such a way that the gain of a hydrogen bond with Ser126 may compensate the loss of the quadrupole–quadrupole interaction with Phe105.

**Figure 8:**
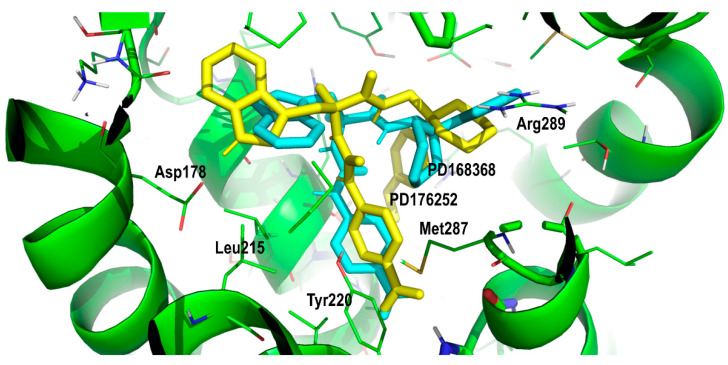
Superposition of PD168368 (cyan) and PD176252 (yellow) in their prospective bound conformations, respectively, to the BB1R.

According to the previous analysis, we can propose prospective conformations of PD168368 and PD176252 bound to the BB1R. Figure 8 shows pictorially the two compounds superimposed when bound to the orthosteric site. Inspection of Figure 8 shows the nitrophenyl group sitting close to Tyr220 and His283 and the indole moiety sitting in a hydrophobic pocket defined by Phe181, Pro120 and Leu215 for both ligands, as described above. These residues were all conserved in the BB2R, so it is not expected that they can provide an explanation for the affinity differences between BB1R and BB2R. On the other hand, the 2-pyridincyclohexane moiety apparently bound in a different conformation in the two ligands. In the case of PD168368, the pyridine ring adopted an extended conformation, whereas in the case of the PD176252, the corresponding dihedral angle was twisted to 60°. This differential conformation permitted the pryridine ring of PD176252 to access a hydrophobic pocket and form a hydrogen bond with the side chain of Ser126, as explained above. In contrast, PD168368 adopted a conformation that permitted the formation of a hydrogen bond with Arg289 together with a quadrupole–quadrupole interaction with Phe105.

In order to further assess the feasibility of both prospective bound conformations, we checked the extent that these models explained the available structure–activity results of this family of compounds [18]. Analysis of the activity of the diverse compounds showed that when the nitro group of the nitrophenyl moiety was attached in position 3 or when it was substituted by a proton accepting chemical group such as nitrile, the compound preserved its activity. However, when the nitro group was placed in position 2 or substituted by a group with lower proton accepting capability, the affinity dropped at least one order of magnitude. The same trends were observed regarding the binding affinity for the BB2R. On the other hand, substitutions on the 2-pydirydine moiety that preserved a proton accepting center in position 4 had similar behavior.

### Proof of Concept

**Figure 9:**
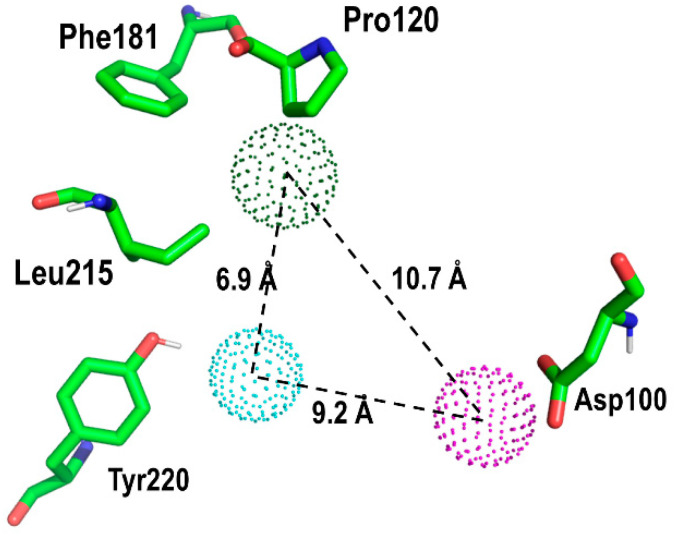
3-point pharmacophore defined by the geometries of a few residues characterizing the sterochemical features involved in BB1R binding (see text).

Using the prospective bound conformation of PD176252 onto the BB1R, we proceeded to identify those interactions that appeared to be key for ligand–receptor recognition, aimed at defining a pharmacophore that could be used as a query for an in silico virtual screening. Accordingly, a simple three-point pharmacophore was defined as shown in Figure 9. The pharmacophore was defined as simply as possible to identify hits with chemical scaffolds of diverse profiles. The pharmacophore involved Tyr220 in the form of a proton accepting center, aimed at discovering BB1R selective compounds, since the BB2R does not have this capability. Specifically, the 3-point pharmacophore included: (i) a proton accepting center in the direction of the OH bond of the hydroxyl moiety of Tyr220 side chain, located at 2.5 Å of the hydroxyl hydrogen; (ii) a hydrophobic center located at a point defined by the side chains of Pro200, Phe181 and Leu215; (iii) a proton donor center located in the plane defined by the atoms of the carboxyl group of Asp100 at 3.0 Å from the center of the two oxygens.

The three-point pharmacophore was used as a query for an in silico screening of diverse databases using the Molecular Operating Environment (MOE) program [32]. More precisely, the query was defined in the form of three spheres with diverse radii to introduce some tolerance in each of the pharmacophoric points. Specifically, the proton acceptor and donor spheres were defined with a 0.12 nm radius, whereas the hydrophobic was defined within a 0.18 nm radius. Two databases of 3D structures of small molecules were used for the screening process including the leadlike database included in the MOE software containing around 650,000 commercially available compounds [32] and the leads-now subset of the ZINC database containing approximately 4,200,000 unique molecules downloaded in 2015 [33]. For each compound, in addition to its 3D structure, the database includes a set of conformations generated using a build-up procedure from systematic conformational searches of molecular fragments.

After screening the 3D databases, a few hundred hits were identified and subjected to a diversity analysis [34]. For this purpose, molecules were encoded as bit strings using the typed atom triangle (TAT) methodology in which atoms are grouped in trios based on information about their chemical nature and mutual distance [35]. In a next step, the distance between bit strings was computed using the Tanimoto coefficient [36]. Finally, molecules were grouped in clusters using the Jarvis-Patrick algorithm [37]. This procedure permitted us to select a subset of compounds preserving the diversity of the initial set, showing the diversity of chemical scaffolds. Representative molecules of the diverse clusters were selected according to the suitability of chemical groups responsible for each of the pharmacophoric points and checking that molecules may not suffer steric clashes. A set of about fifty selected compounds were docked onto the BB1R using the GLIDE software [29] and ranked order according to the XP scoring function. Thirteen compounds among those with the best score were purchased (Appendix A) and tested at 50 µM for their capacity to displace the radioligand to the BB1R, as explained in the methods section [38]. The chemical structures of the six compounds were found to displace the radioligand used in the binding assays by more than 15%, as listed in Table 1. These results yielded a success rate of about 50%, as found in other cases [39,40]. Table 1 also lists the results of radioligand displacement experiments for the BB1R and BB2R, suggesting that the ligands are BB1R selective.

**Figure 10:**
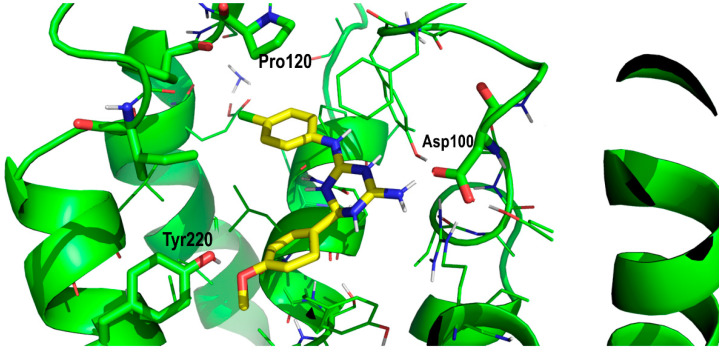
Pictorial view of the proposed binding mode of Compound #**5** to the bombesin BB1 receptor.

In order to explain these results, we proceeded to dock the novel hits disclosed onto the BB1R following the same protocol as explained in the methods section. As an example, Figure 10 shows pictorially compound #**5** in its prospective bound conformation to the BB1R. As can be seen, the triazine ring works as a scaffold with three branches: a phenoxyl moiety, a chlorophenyl moiety and an amine. Analysis of the prospective bound conformation showed that the oxygen of the methoxyl group fulfilled pharmacophoric point #**1**, exhibiting a hydrogen bond interaction with the hydroxyl moiety of Tyr220. Moreover, the chlorine atom sat at the hydrophobic site (pharmacophoric point #**2**) and the amine fulfilled pharmacophoric point #**3** exhibiting a hydrogen bond with Asp300. In the case of the BB2R, these ligands could not attain the hydrogen bond with Tyr220, fulfilling only two pharmacophoric points, explaining that their affinity for the BB2R is much lower than for the BB1R.

Finally, although the affinities exhibited by the hits presently disclosed is not very high, they represent a set of small molecules with high diversity that can be exploited further to discover novel selective antagonists for the BB1R.

## 3. Materials and Methods

### 3.1. Molecular Modeling

A crude model of the human bombesin BB1 receptor was constructed by homology modeling using the rat neurotensin receptor NTS1 (PDB entry: 4GRV) as a template [41]. The template was selected for being one of the few receptors with a known crystallography structure located in the same branch of bombesin in the GPCRs phylogenetic tree [28,42]. Since the 4GRV structure corresponds to a fusion protein of NTS1 and the T4 Lysozyme, the template structure was edited by removing the coordinates of the latter and joining the segments of the ECL3 left at both sides. In a subsequent step, the sequences of the BB1 and the NST1 receptors were aligned. This process is critical for ensuring the accuracy of the models constructed by homology modeling [43]. Since in this case, sequence identity is low (~23%; 43% sequence similarity), we undertook a multiple sequence alignment to improve its quality [44]. Accordingly, we selected a set of 20 sequences of diverse GPCRs of the class-A with a known crystallographic structure plus the three bombesin receptors that were aligned using the CLUSTALW software (version 2.0.12) [45]. To finalize the alignment process, the two sequences of interest were checked to ensure the alignment of diverse known conserved residues in the class A family of GPCRs and motifs such as D(E)RY in TM3, CWxP(Y/F/L) in TM6 or the NPxxY in TM7, as well as the location of the conserved disulfide bridge between ECL2 and TM3. In a further step, the alignment was used to thread the sequence of the BB1 receptor onto the backbone of the template structure, using the Molecular Operating Environment (MOE) program (version 2019.01) [32]. Thirty models were produced after the incorporation of alternative sidechain conformations, using an extensive rotamer library embedded in MOE, generated from a high-resolution structural database. Once hydrogens were added to the models using the protonate3D method [46], each of them was energy minimized using a contact energy function to relieve any serious steric strains. Models were checked for inter-residue contacts as well as for backbone conformations through the Ramachandran map and scored. Finally, the model with the lower root-mean square deviation (rmsd) in regard to the average structure with the highest score was selected as a crude model and considered for refinement.

Refinement of the receptor was carried out using molecular dynamics with a ligand bound in the orthosteric site. The presence of a ligand provides a more efficient refinement of the constructed model [28]. Accordingly, the antagonist PD176252 (Figure 1) [17] was docked in different conformations into the orthosteric site of the crude model using the GLIDE software (version 6.7) [29]. Multiple orientations were obtained that were rank ordered using the XP scoring function. The pose with the highest score was energy minimized in vacuo with a distance dependent dielectric constant of **2** using the steepest descent method to permit a reorientation of the ligand. Subsequently, the ligand–receptor complex was embedded in a lipid bilayer. Specifically, the protein was embedded in a box consisting of a 1-palmitoyl-2-oleoyl-sn-glycero-3-phosphocholine (POPC) lipid and water molecules generated and equilibrated according to the procedure described previously [47]. The box had an initial size of 8.9 × 8.3 × 10.5 nm^3^ (XYZ), organized in such a way that the bilayer plane was oriented on the XY plane. The protein was placed in the center of the box, and the overlapping molecules were removed. Specifically, all water molecules with oxygen atoms closer than 0.40 nm to a nonhydrogen atom of the protein, as well as all lipid molecules with at least one atom closer than 0.25 nm to a nonhydrogen atom of the protein, were removed. This resulted in a final system containing 193 lipids and ca. 12,000 water molecules. Removal of these atoms introduced small voids between the protein and water or lipid molecules that disappeared during the first part of the molecular dynamics (MD) simulation, in which a progressive adjustment of the lipid bilayer and water molecules to the protein takes place. Next, 101 randomly selected water molecules were replaced by 47 sodium and 54 chloride ions, providing a neutral system with a concentration approximately 0.2 M in sodium chloride. This concentration is similar to that found in biological organisms, although they exhibit different intra- and extracellular ion concentrations. Then, the system was energy minimized to avoid steric classes using the steepest descent method and subjected to a 500 ns MD simulation at a constant pressure using the GROMACS package 4.6 [48]. Molecules were described using the all-atom OPLS force field [49] currently implemented in GROMACS, except for water molecules that were modeled using the TIP3P model [50]. The system was subjected to periodic boundary conditions in the three coordinate directions. The temperature was kept constant at 300 K using separate thermostats for the protein, water, ions and lipid molecules. The time constant for the thermostats was set to 0.1 ps, except for water, for which a smaller value of 0.01 ps was used. The pressure in the three coordinate directions was kept at 0.1 MPa by independent Berendsen barostats using a time constant of 1.0 ps. The equations of motion were integrated using the leapfrog algorithm with an integration step of **2** fs. All bonds involving hydrogen atoms within the protein and lipid molecules were kept frozen using the LINCS algorithm [51]. The bonds and the angle of water molecules were fixed using the analytical SETTLE method. Lennard–Jones interactions were computed using a cutoff of 1.0 nm. Electrostatic interactions were treated using the particle-mesh Ewald procedure [52].

### 3.2. Binding Assays

BB1 antagonism assays were carried out following a protocol described elsewhere [42]. Specifically, human recombinant bombesin BB1 receptors expressed in CHO-K1 cells were used in modified HEPES-KOH buffer pH 7.4 (Thermo Scientific, Waltham, MA, USA). A 0.2 μg protein aliquot was incubated with 0.1 nM [^125^I][Tyr^4^]-bombesin for 60 min at 25 °C. Nonspecific binding was estimated in the presence of 1 μM neuromedin B. Membranes were filtered and washed; the filters were then counted to determine [^125^I][Tyr^4^]-bombesin (K_d_ = 0.13 nM) specifically bound. Hits were screened at 50 μM. Compound binding was calculated as the percentage of the inhibition of the binding of a radioactively labeled ligand.

## 4. Conclusions

This paper reports the results of a modeling study aimed at shedding some light on the stereochemical requirements for small molecule binders to the BB1 bombesin receptor. For this purpose, we first constructed a 3D model of the bombesin BB1 receptor by homology modeling using the rat neurotensin receptor as a template. Then, the model was refined using molecular dynamics in a system composed by the receptor embedded in a bilayer of POCP lipids, water and sodium chloride. The MD simulation was carried out with the ligand PD176252 bound to the receptor in its orthosteric site to accelerate the refinement process. After 500 ns sampling, the refined structure of the receptor was computed as the average of the diverse configurations sampled during the last 100 ns of the trajectory. Subsequently, the modeled 3D structure of the receptor was used to dock the antagonists PD168368 and PD176252 in its orthosteric site. Analysis of the complexes, guided by structure–activity and mutagenesis studies available, permitted us to propose prospective complexes of the bound conformation of each of the ligands to the BB1 receptor. The results of this study directly connect diverse pieces of information that were available in the literature and can be used as the basis for designing new experiments and small molecule ligands.

As a proof of principle, we also carried out an in silico screening using a simple pharmacophore defined from the complex of PD176252 bound to the BB1R. Specifically, a three-point pharmacophore that involves a point exclusive for the BB1 receptor was used for this purpose. The in silico study permitted us to identify a set of small molecules with affinity for the BB1 receptor that were also disclosed. Interestingly, none of the molecules exhibited affinity for the BB2 receptor. The set of molecules have scaffolds of a diverse chemical nature that can be used as a starting point for the development of novel BB1 antagonists.

## Figures and Tables

**Table 1:** Listing of small molecules identified in the in silico screening (see text). Column 2 shows their chemical structure and columns 3 and 4 the displacements of the corresponding BB1R and BB2R radioligands, respectively, (in percentage) at 50 µM (*n* = 2).

Compound#	Chemical Structure	BB1R(Neuromendin B Receptor) Radioligand Displacement (%)	BB2R(Gastrin-Releasing Peptide Receptor) Radioligand Displacement (%)
1	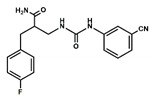	19.7	0.0
2	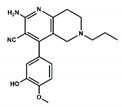	24.3	0.0
3	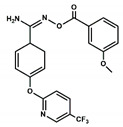	28.1	0.0
4	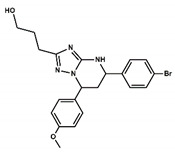	30.5	0.0
5	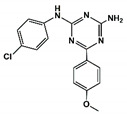	38.0	10.5
6	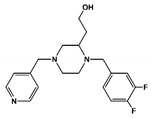	16.1	0.0

## Appendix A

Catalog Numbers and Suppliers of the Compounds Listed in Table 1.

Compound #1: AKOS000796000 (AKos GmbH, Stuttgart, Germany); Compound #2: Amb19684292 (Ambinter c/o Greenpharma SAS, Orleans, France); Compound #3: CB358 (Menai Organics Ltd., Bangor, UK); Compound #4: Amb1220897 (Ambinter c/o Greenpharma SAS, Orleans, France); Compound #5: Amb3992353 (Ambinter c/o Greenpharma SAS, Orleans, France); Compound #6: Amb11089933 (Ambinter c/o Greenpharma SAS, Orleans, France).
